# Phenotypic Landscape of Programmed Death and TIGIT/CD155/DNAM-1 Pathways in NK Cells in Ovarian Cancer

**DOI:** 10.3390/biomedicines14102400

**Published:** 2026-10-21

**Authors:** Anna Pawłowska-Łachut, Wiktoria Skiba, Paulina Pieniądz-Feculak, Karolina Włodarczyk-Ciekańska, Dorota Suszczyk, Jakub Łyczba, Monika Bilska, Michalina Kazimierczuk, Józef Kotarski, Ewa Boniewska-Bernacka, Jacek Tomaszewski, Iwona Wertel

**Affiliations:** 1 Independent Laboratory of Cancer Diagnostics and Immunology, Medical University of Lublin, Chodźki 1, 20-093 Lublin, Poland; 2 Department of Virology and Immunology, Institute of Biological Sciences, Maria Curie-Skłodowska University, Akademicka 19, 20-033 Lublin, Poland; 3 Second Department of Gynecological Oncology, St. John’s Center of Oncology of the Lublin Region, 20-090 Lublin, Poland; 4 Department of Biology and Genetics, Institute of Medical Sciences, University of Opole, Oleska 48, 45-052 Opole, Poland

**Keywords:** ovarian cancer, TIGIT, PD-1, tumor microenvironment, dual blockade, DNAM-1

## Abstract

**Objectives**: Ovarian cancer (OC) is the gynecological malignancy with the highest mortality rate. Preclinical studies have shown that dual blockade of immune checkpoint proteins (ICPs), such as PD-1 and TIGIT, may be beneficial for OC patients. Thus, the aim of this study was to evaluate the expression of PD-1, TIGIT, and DNAM-1 in NK cells in three OC environments—i.e., peripheral blood (PB), peritoneal fluid (PF), and tumor tissue (TT)—and their soluble forms in plasma and PF to explore their clinical significance. **Methods**: The expression of ICPs on NK cells was analyzed via flow cytometry. The concentrations of soluble forms of these ICPs were measured in plasma and PF via ELISA. The obtained results were correlated with the clinicopathological features of OC patients. **Results**: We observed an increased percentage of PD-1^+^ NK cells in tumor tissue (*p* < 0.05), as well as TIGIT^+^ (*p* < 0.001) and DNAM-1^+^ NK cells in PF of patients with OC (*p* < 0.05). Soluble PD-1 (sPD-1) concentration was higher in PF than in plasma (*p* < 0.0001) and higher in plasma of OC patients than in controls (*p* < 0.001), while soluble TIGIT (sTIGIT) levels were elevated in plasma of OC patients compared with controls (*p* < 0.05). NK cell phenotypic alterations were also associated with selected clinicopathological features of OC (i.e., FIGO disease advancement, tumor type according to Kurman and Shih, and grade). The percentage of NK cells in PF was positively associated with age (*p* < 0.01) and negatively associated with BMI (*p* < 0.05). **Conclusions**: OC is characterized by distinct compartment-specific patterns of PD-1, TIGIT, and DNAM-1 expression on NK cells, accompanied by altered soluble ICP profiles. These phenotypic patterns may help define the immune landscape of the ovarian cancer microenvironment.

## 1. Introduction

Ovarian cancer (OC) is considered the most lethal gynecological malignancy. This is related to its diagnosis in advanced International Federation of Gynecology and Obstetrics (FIGO) stages of the disease as a result of the disease heterogeneity, lack of screening tools, and an asymptomatic course of the disease, especially in the early stages [1,2,3]. Notably, OC in early FIGO stages (stages I and II) is curable. Nonetheless, the majority of cases (approximately 85%) are diagnosed in advanced stages (FIGO stages III and IV) when the five-year survival rate decreases to 29.2%. By way of comparison, for local diseases (FIGO stages I and II), the five-year survival rate is 92% [4,5], and the total five-year survival rate is as low as 47% [6]. Despite the implementation of standard treatments, including cytoreductive surgery, chemotherapy, and biological drugs—such as vascular endothelial growth factor inhibitors (VEGFis; e.g., bevacizumab) and poly(ADP-ribose) polymerase (PARP) inhibitors (PARPis; e.g., olaparib, rucaparib, and niraparib)—the prognosis for OC patients is poor [7,8]. Thus, there is a need to develop diagnostic/prognostic biomarkers and novel treatment strategies for OC patients.

The dynamic and complex interplay within the tumor microenvironment (TME) is a potential target for OC treatment [5]. Immune cells are influenced by various signals from the TME that shape their immunophenotype and modulate their functions. The immunogenicity of cancer directly affects immune evasion through multiple mechanisms, such as immune checkpoint proteins (ICPs), and their expression is considered a crucial factor in predicting the response to immune checkpoint inhibitor (ICI) therapy [9].

In the previous decade, immunotherapies based on immune checkpoint inhibitors, including programmed death pathway inhibitors, revolutionized the treatment of solid malignancies such as melanoma and lung and renal cancer [10,11,12]. This type of treatment has also been approved for the treatment of gynecological malignancies such as uterine and cervical cancer [13]. Nevertheless, inhibitors of programmed death receptor 1 (PD-1) (e.g., nivolumab and pembrolizumab), as well as programmed death ligand 1 (PD-L1) inhibitors (e.g., atezolizumab, avelumab, and durvalumab), have shown limited efficacy in OC treatment [5,14]. The response rate of OC patients to these drugs is low to intermediate [15,16], with a total response rate of 10–15%. Notably, the resistance of OC to ICIs is related to a number of factors, such as a decreased density of TILs, OC heterogeneity, low microsatellite instability, tumor mutation burden, regulation of ICPs via cytokine and miRNA networks, and interplay within the TME. The complexity of the molecular, genetic, and immunological background of OC patients resistant to ICIs was presented in detail in our previous paper [14].

The Food and Drug Administration (FDA) has not approved any ICIs for the treatment of OC monotherapy [8]. On February 10, 2026, the FDA approved pembrolizumab in combination with paclitaxel, with or without bevacizumab, for adults with PD-L1-positive platinum-resistant ovarian cancer after one or two prior therapies. This finding represents a key advance in the introduction of biomarker-selected immunotherapy into the earlier management of platinum-resistant OC [17].

Moreover, recent studies have shown that focusing only on the PD-1/PD-L1/PD-L2 pathway, its inhibitors, and T cell activity with respect to immunosuppression in the ovarian TME is insufficient [5,18,19,20,21,22]. To fill this gap, more comprehensive studies are needed on the complexity of interactions in the TME, including an understanding of the activity of other effector cells and coexpressed ICPs in terms of clinical implications.

Although the PD-1 receptor is considered a main regulator of T cell activity, other coexpressed ICPs, such as costimulatory DNAX accessory molecule-1 (CD226; DNAM-1) and coinhibitory T cell immunoglobulin and the ITIM domain (TIGIT), influence their functions [23,24,25]. Both TIGIT and PD-1 receptors are exhaustion markers of cytotoxic T cells (CD8^+^) [26].

**Figure 1:**
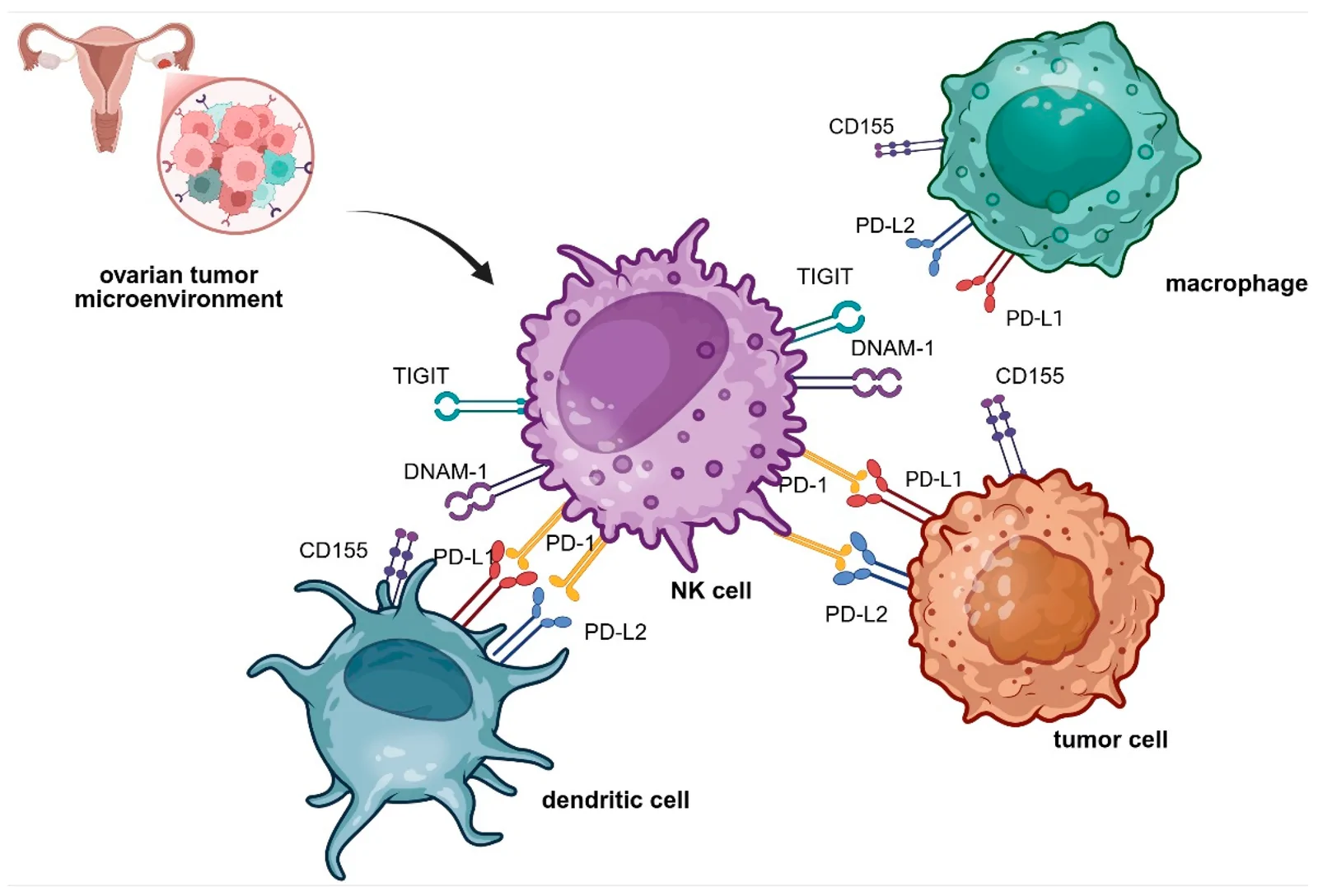
Interactions between the TIGIT/CD155/DNAM-1 and PD-1/PD-L1/PD-L2 pathways. Created in BioRender. Pawłowska-Łachut, A. (2026) https://BioRender.com/ya9o0z7.

The interplay between ligands and receptors in the TIGIT/CD155/DNAM-1 axis is far more intricate than that in the PD-1/PD-L1/PD-L2 pathway. Among the ligands for TIGIT are CD155, CD112, CD113, and nectin-4. The complexity of the axis is emphasized by the fact that the ligands CD155 and CD112 are shared with the costimulatory receptor DNAM-1 and the inhibitory receptor TACTILE (CD96). The detailed information concerning the interaction between the PD-1/PD-L1/PD-L2 pathway and the TIGIT/CD155/DNAM-1 axis and the effects of dual blockade in OC patients was provided in our previous review article [27]. The complex network of interactions between the TIGIT/CD155/DNAM-1 and PD-1/PD-L1/PD-L2 pathways is presented in Figure 1.

Recent studies have shown that the implementation of dual blockade of ICPs, especially the combination of PD-1/PD-L1 and TIGIT inhibitors, may be beneficial for OC patients. The binding of TIGIT to its ligands results in the inhibition of NK cell and T cell activity, proliferation, and cytokine secretion [27]. Unlike TIGIT, DNAM-1 is a costimulatory receptor, and its binding to CD115 or CD112 stimulates the lysis activity of NK cells targeted against cancer cells. Nonetheless, the ligand CD155 has a lower affinity for DNAM-1 than for TIGIT [23,24,25,28,29,30].

Importantly, both pathways work synergistically. Mechanistically, both pathways share a common factor, the costimulatory receptor DNAM-1. Both PD-1 and TIGIT inhibit the activity of DNAM-1 [23,24,25,26]. Therefore, both pathways need to be simultaneously blocked to fully restore DNAM-1 costimulatory signaling. These findings highlight the reasons why dual blockade has more promising clinical outcomes [26,31,32,33].

The crosstalk between PD-1, TIGIT, and DNAM-1 on NK cells in OC patients, considering their impact on immune regulation and immunosuppression within the tumor microenvironment, is a potential target for precision medicine. These ICPs modulate the activity of NK cells, influencing their ability to recognize and eliminate tumor cells, thus offering potential therapeutic targets for enhancing antitumor immunity.

Here, we comprehensively evaluated the expression of the main receptors involved in the programmed death pathway and the TIGIT/CD155/DNAM-1 axis—i.e., PD-1, DNAM-1, and TIGIT—in NK cells in peripheral blood (PB), peritoneal fluid (PF), and tumor tissue (TT) with respect to their clinical implications in OC patients. We also evaluated the concentrations of soluble forms of these receptors—i.e., soluble PD-1 (sPD-1), sDNAM-1, and sTIGIT—in the plasma and peritoneal fluid of OC patients compared with those in the plasma of healthy blood donors. The obtained results were analyzed in relation to the clinicopathological data of patients in comparison to those of healthy blood donors to investigate their clinical implications.

## 2. Materials and Methods

### 2.1. Patient Enrollment and Ethics Statement

The research involved 56 OC patients and 20 healthy blood donors. OC patients had undergone radical treatment at the II Department of Gynecological Oncology (St. John’s Center of Oncology of the Lublin Region, Lublin). All samples were processed fresh on the day of surgery, immediately after collection. Samples were collected over the study period from 2024 to 2026. The histopathological reports confirmed the diagnosis of OC. The tumors were staged in accordance with the FIGO classification system [34]. Two independent gynecological pathologists classified and graded the tumors on the basis of the Silverberg grading system [35], and the tumor type was established in accordance with the Kurman and Shih classification [36,37]. The study cohort excluded individuals with a history of previous malignancies; prior chemotherapy or radiation therapy; and allergic, infectious, or autoimmune diseases. The clinicopathological characteristics of the OC patients are shown in Table 1.

Peripheral blood samples were obtained from healthy female blood donors (*n* = 20) at the Regional Centre for Blood Donation and Blood Therapy in Lublin, Poland. The healthy donor cohort had a median age of 47.5 years (range 43–52 years) and a median BMI of 24.06 kg/m^2^ (range 22.14–26.45 kg/m^2^). All donors were female and were premenopausal.

The study was conducted in accordance with the Declaration of Helsinki and approved by the Institutional Review Board of Medical University of Lublin (KE-0254/13/01/2022; KE-0254/102/06/2024; KB-0024/178/12/2024; KB-0024/114/01/2026). Informed consent was obtained from all subjects involved in the study.

### 2.2. Plasma and Mononuclear Cell (MNC) Isolation Procedure

All PB samples were drawn into EDTA-coated tubes (Sarstedt, Germany; cat. no. I-5729) prior to surgery and processed immediately. Samples were centrifuged at 1000× rpm for 15 min at room temperature to separate plasma from cellular components. The upper plasma layer was aliquoted into clean 1.5 mL Eppendorf tubes and stored at −80 °C until further analysis. MNCs were isolated via density gradient centrifugation at 2800× rpm for 20 min at room temperature using Lymphoprep (StemCell Technologies, Vancouver, BC, Canada). The cells from the interphase layer were then collected. After two washes with phosphate-buffered saline (PBS; Capricorn Scientific GmbH, Ebsdorfergrund, Germany; cat. no. PBS-1A), the cells were resuspended in PBS.

During surgery, PF and TT were collected aseptically. MNCs from PF were also isolated via centrifugation in a density gradient (2800× rpm for 20 min at room temperature) using Lymphoprep (StemCell Technologies, Vancouver, BC, Canada; cat. no. 18060). The following procedure was the same as that used for MNCs isolated from PB.

Freshly resected OC tissue was chopped and placed in a gentleMACS C tube (Miltenyi Biotec, Bergisch Gladbach, Germany; cat. no. 130-093-237) and then processed via the Tumor Dissociation Kit (Miltenyi Biotec; cat. no. 130-095-929) to isolate tumor-infiltrating MNCs. The suspensions of cells were subsequently filtered with a mesh filter (70 mm; BD Biosciences, Milpitas, CA, USA, cat. no. 340635), and the subsequent procedure, including density gradient centrifugation and washing, was carried out in the same manner as described for the PB and PF samples.

### 2.3. NK Cell Immunophenotyping via Flow Cytometry

All samples were processed fresh on the day of surgery, immediately after collection. Samples were collected over the study period from 2022 to 2026. Tumor samples (PB, PF, and TT) originated from the same patients whenever possible.

**Figure 2:**
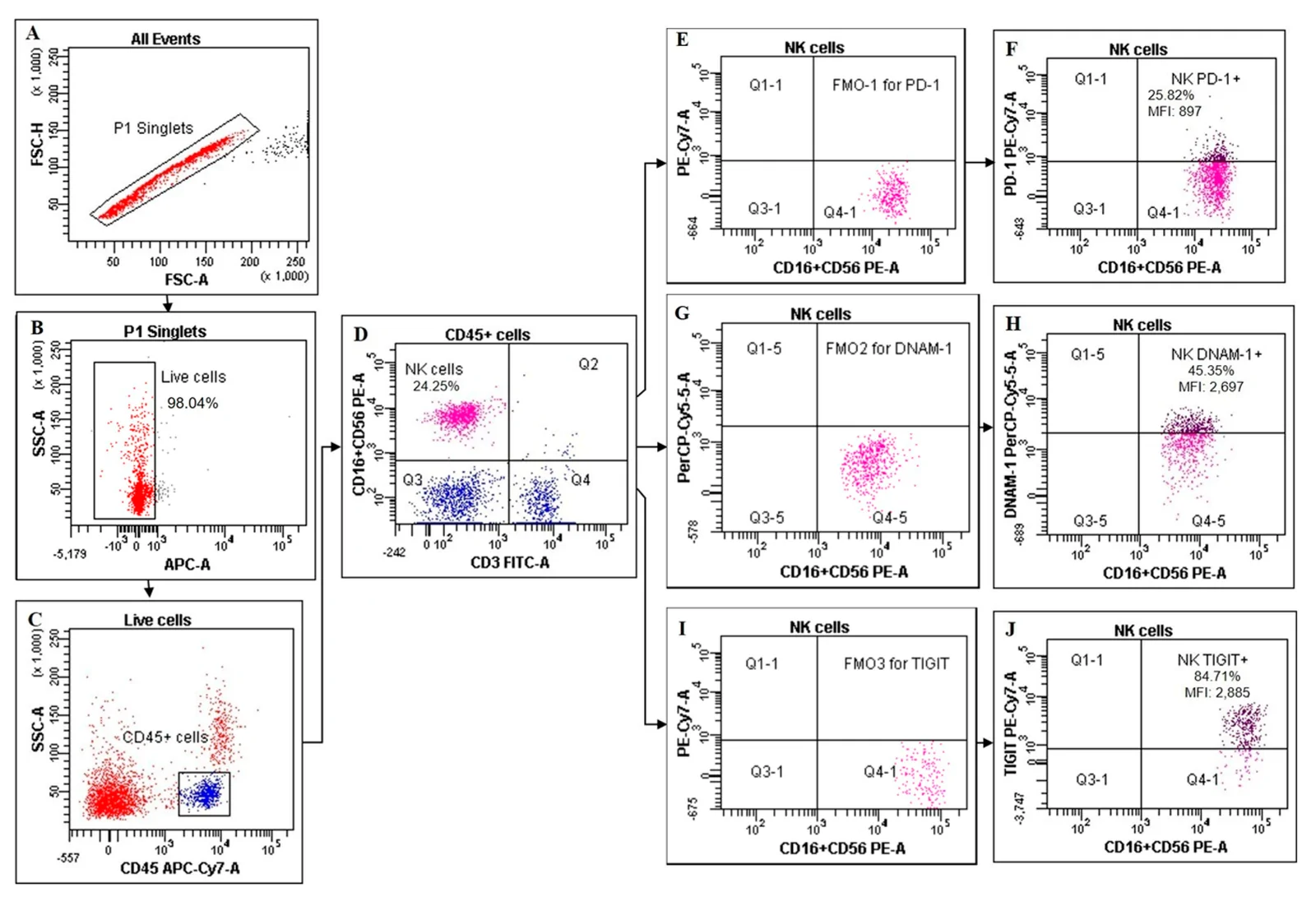
The gating strategy for NK cells with PD-1, TIGIT, or DNAM-1 expression. NK cells were identified with the following gating strategy: doublets were excluded with a combination of forward scatter area (FSC-A) and forward scatter height (FSC-H) (P1 Singlets) (**A**). Live cells analyzed for fluorescent reactive dye staining were selected from the P1 Singlets (LIVE/DEAD Fixable Aqua Dead Cell Stain Kit, Fixable Near-IR Dead Cell Stain Kit, 633/635 nm excitation [APC], Thermo Fisher Scientific, Waltham, MA, USA) (**B**). CD45^+^ leukocytes were selected from the live cell population (**C**). NK cells were identified within CD45^+^ leukocytes as CD16^+^CD56^+^CD3^−^ cells (**D**). Selected CD16^+^CD56^+^CD3^−^ cells were analyzed for PD-1, DNAM-1, and TIGIT expression. The final dot plots indicate NK cells positive for PD-1 (**F**), TIGIT (**H**), and DNAM-1 (**J**). Dot plot (**E**) is the FMO control for PD-1 (FMO1), stained with all markers in the panel except for PD-1. Dot plot (**G**) is the FMO control for DNAM-1 (FMO2), stained with all markers in the panel except for DNAM-1. Dot plot (**I**) is the FMO control for TIGIT (FMO3), stained with all markers in the panel except for TIGIT.

To assess cell viability, MNCs (1 × 10^6^ cells) isolated from PB, PF, and TT were incubated with aqua fluorescent reactive dye (LIVE/DEAD Fixable Aqua Dead Cell Stain Kit, Fixable Near-IR Dead Cell Stain Kit, for 633 or 635 nm excitation [APC]; Thermo Fisher Scientific, Waltham, MA, USA; cat. no. L34976) according to the manufacturer’s instructions (Figure 2B). Then, MNCs were stained with fluorochrome-labeled monoclonal antibodies (mAbs) directed against specific surface markers via incubation of MNCs with appropriate mAbs for 20 min. The panel of fluorochrome-conjugated monoclonal antibodies included the following: CD45 APC-H7 clone 2D1 (BD Biosciences, cat. no. 560178; 5 µL per test), anti-CD3 FITC/(CD16 + CD56) PE Cocktail clone UCHT-1 (BioLegend, San Diego, CA, USA; cat. no. 319101; 20 µL per test), anti-PD-1 PE/Cyanine7 clone A17188B (BioLegend, cat. no. 621608, 5 µL per test), anti-TIGIT PE/Cyanine7 clone A15153G (BioLegend, cat. no. 372704; 5 µL per test), and anti-DNAM-1 PerCP/Cyanine5.5 clone 11A8 (BioLegend, cat. no. 338314; 5 µL per test). After incubation in the dark for 30 min (at room temperature, RT) and washing in 1% bovine serum albumin-enriched PBS. Samples with a cell viability above 95% were included in the flow cytometric analysis (FACSCanto I, Becton Dickinson, Franklin Lakes, NJ, USA). For each sample, 50,000 events were recorded.

The FacsDiva software (v6.1.3) was used to determine the percentage of NK cells and NK cells expressing PD-1, TIGIT, or DNAM-1. The percentages of NK cells with ICPs expression were reported as percentages relative to each specific cell subset (i.e., PD-1^+^CD56^+^CD16^+^CD3^−^ cells, TIGIT^+^CD56^+^CD16^+^CD3^−^ cells, or DNAM-1^+^CD56^+^CD16^+^CD3^−^ cells populations). The gate for PD-1^+^, TIGIT^+^, DNAM-1^+^ cells was set based on the fluorescence minus one (FMO) control. FMO control contained all fluorochromes in a panel except for PD-1^+^, TIGIT^+^, and DNAM-1^+^. The FMO controls were applied to identify any spread of fluorochrome in an unlabeled channel and to assist in accurate gate placement when defining positive cell populations, and were used to establish appropriate gating thresholds. The representative FMO controls for PD-1^+^, TIGIT^+^, and DNAM-1^+^ are provided in Figure 2.

### 2.4. Enzyme-Linked Immunosorbent Assay Procedure

The concentrations of soluble forms of the selected ICPs—i.e., sPD-1, sTIGIT, and sDNAM-1—in the paired plasma and PF obtained from the same OC patients and the plasma of healthy blood donors were established via ELISA tests according to the manufacturer’s instructions (sPD-1, Thermo Fisher Scientific, Waltham, MA, USA; cat. no. BMS2214 [intra-assay CV% < 3.2%, inter-assay CV% < 6.4%, range: 2.34–150.0 pg/mL; dilution: 2×]; sTIGIT, ELK Co., Ltd. Biotechnology, Guangzhou, China; cat. no. ELK7357 [intra-assay CV% < 8%, inter-assay CV% < 10%, range: 0.32–20 ng/mL; dilution: 1×]; sDNAM-1, Biorbyt, Cambridge, UK; cat. no. orb564954 [intra-assay CV% < 5.16%, inter-assay CV% < 4.69%, range: 78.125–5000 pg/mL; dilution: 2×]).

The absorbance of the plate was measured via an ELX800 plate reader (BioTek Instruments, Inc., Winooski, VT, USA). The obtained results were analyzed with Gen5 software (version 2.09) (BioTek Instruments, Inc.). The concentrations of sPD-1, sTIGIT, and sDNAM-1 were determined via interpolation from a standard curve. Values below the lower limit of detection (LLOD) were treated as missing values and excluded from statistical analyses. No numerical value, including zero, was assigned to these measurements.

### 2.5. Statistical Analysis of the Obtained Results

The obtained results were statistically analyzed via GraphPad Prism (9.1.2) and Statistica (13.0PL). The normality of data distribution was assessed using the Shapiro–Wilk test, which indicated a non-normal distribution of the data. To compare the results from paired samples—i.e., PB, PF, and TT within the same OC patients—the Wilcoxon paired test was used. For comparisons between groups (e.g., study group vs. control group), the Mann–Whitney U test was applied. To assess the correlation between two variables, the Spearman rank correlation test was utilized. To account for multiple comparisons, *p*-values were adjusted using the Benjamini–Hochberg (BH) false discovery rate (FDR) correction. The results are presented as the median, with minimum and maximum values. Statistical significance was defined as a *p*-value of less than 0.05 (*p* < 0.05).

## 3. Results

### 3.1. The Percentages of NK Cells in the Peripheral Blood, Peritoneal Fluid, and Among Tumor-Infiltrating Cells from OC Patients

**Figure 3:**
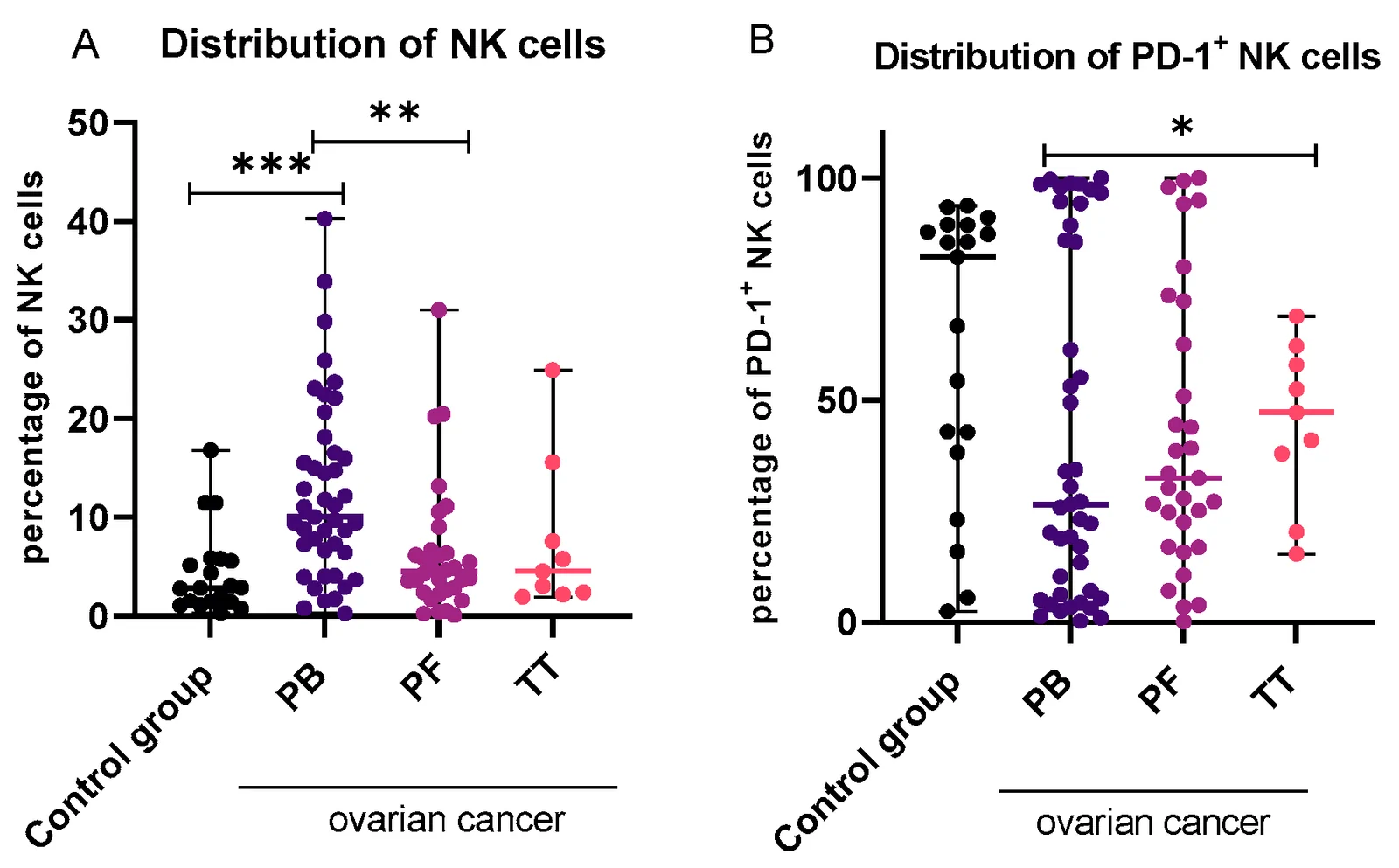

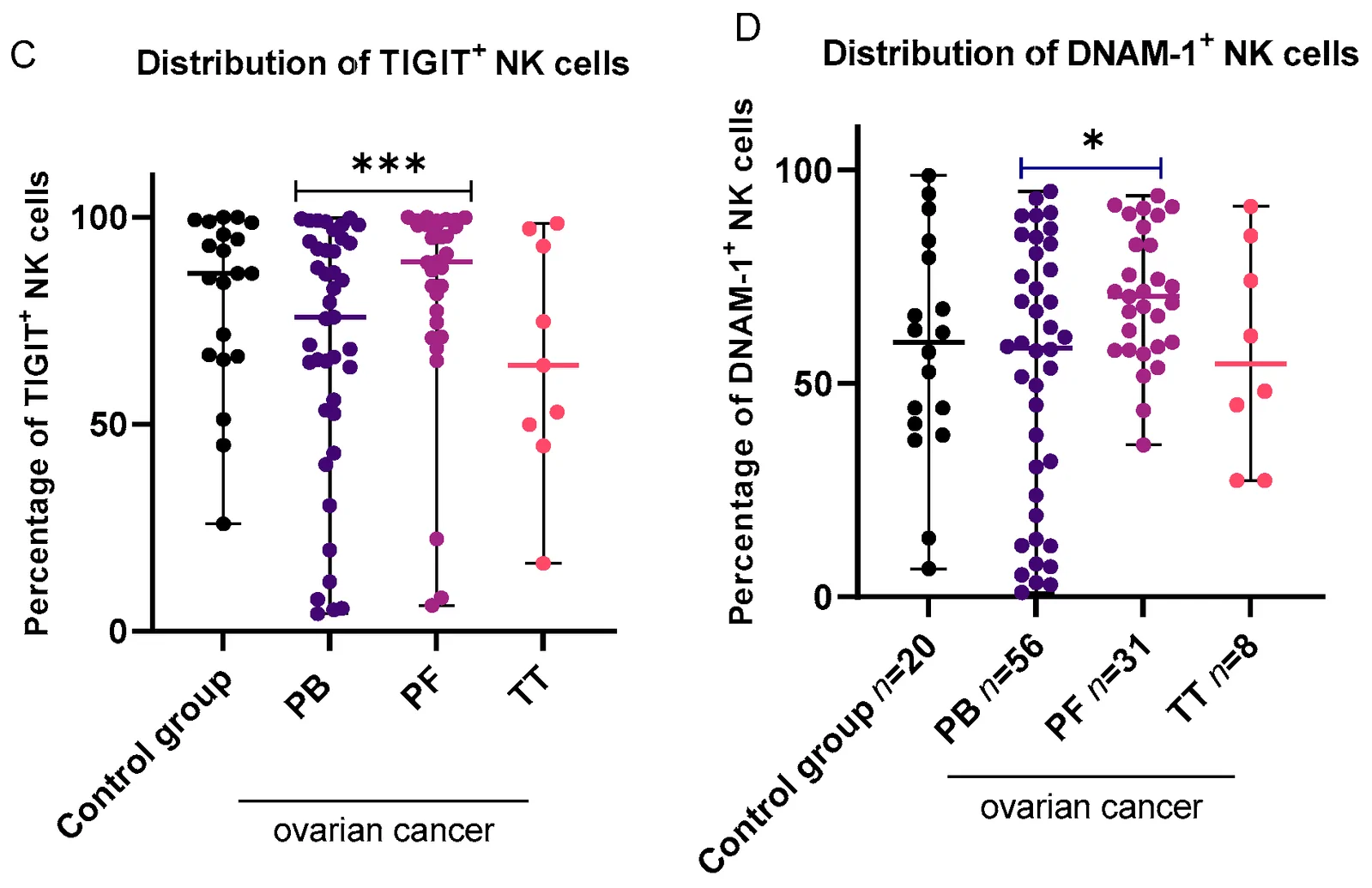
Distribution of NK cells (**A**) and NK (CD56^+^CD16^+^CD3^−^) cells expressing programmed death receptor 1 (PD-1) (**B**), T cell immunoreceptor with Ig and ITIM domains (TIGIT) (**C**), and DNAX accessory molecule-1 (DNAM-1) (**D**) in the peripheral blood (PB), peritoneal fluid (PF), and among tumor-infiltrating cells (TT) of ovarian cancer (OC) patients compared with the PB of healthy donors. Paired PB, PF, and TT samples were compared using the Wilcoxon signed-rank test, while independent groups were compared using the Mann–Whitney U test. Median values with the following marks indicate statistically significant differences: * *p* < 0.05, ** *p* < 0.01, *** *p* < 0.001.

The analysis revealed significant differences in the percentages of CD56^+^CD16^+^CD3^−^ cells between the ovarian cancer environments and the peripheral blood of healthy donors. We observed the highest frequency of CD56^+^CD16^+^CD3^−^ cells in the PB of OC patients than in PF (*p* = 0.001323; BH-adjusted *p* = 0.0053; median 10.05% vs. 4.50%; 95% CI [−9470, −1440]), significantly higher than in the PB of the control group (*p* = 0.000113; BH-adjusted *p* = 0.000791; median 10.05% vs. 2,87%; 95% CI [−10.92, 2.53]), and higher than among OC infiltrating cells; however, the difference did not reach statistical significance (*p* = 0.441269; BH-adjusted *p* = 0.5884; median 10.05% vs. 4.57%; 95% CI [−18.25, 6.80]) (Figure 3A).

The healthy control group consisted exclusively of premenopausal women, whereas the patient cohort included both premenopausal and postmenopausal women, with differences in age and BMI between the groups. These demographic differences should be considered when interpreting comparisons between patients and healthy controls. The lower sample size in the TT group resulted from limited availability of tumor tissue, as in some cases it was not possible to obtain material from every patient or the amount collected was insufficient to perform the analysis.

### 3.2. The Frequency of NK Cells with PD-1, TIGIT, and DNAM-1 Expression in PB, PF, and TT from OC Patients

The obtained data also revealed a disparity between CD56^+^CD16^+^CD3^−^ cells with PD-1, TIGIT, and DNAM-1 expression in different environments of OC patients. As noted above, differences in age, menopausal status, and BMI between healthy controls and patients should be considered when interpreting this comparison. We detected the highest percentage of PD-1^+^CD56^+^CD16^+^CD3^−^ cells among the tumor-infiltrating cells, which was significantly higher than that in PB (*p* = 0.028403; BH-adjusted *p* = 0.0757; median 47.22% vs. 26.57%; 95% CI [−7960, 35.69]) and higher than that in PF (median 47.22% vs. 32.50%; 95% CI [−24.100, 35.36]); nevertheless, the differences were not statistically significant (*p* = 0.092893; BH-adjusted *p* = 0.148629) (Figure 3B). We did not observe a statistically significant difference in the percentage of this cell subpopulation in the PB of patients and the control group (*p* = 0.160098; BH-adjusted *p* = 0.2241; 95% CI [−16.62, 32.79]).

We established the highest frequency of TIGIT^+^CD56^+^CD16^+^CD3^−^ cells in the PF of OC patients, and the value was significantly higher than that in the PB (*p* = 0.000416; BH-adjusted *p* = 0.00333; median 89.18% vs. 75.86%, 95% CI [2580, 18.77]) and higher in comparison to TT (*p*-value and BH-adjusted *p* values total 0.8887; median 89.18% vs. 64.19%; 95% CI [−39.13, 39.82]) and control group (*p* = 0.595640; BH-adjusted *p* = 0.6949; median 89.18% vs. 86.38%; 95% CI [−26.11, 1.84]). Nonetheless, the differences were not significant (*p* > 0.05). We also detected a lower percentage of this cell subpopulation in TT than in the PB of healthy donors (median 64.19% vs. 86.38%; 95% CI [−19.25, 22.19]) (Figure 3C). However, the differences did not reach statistical significance (*p* = 0.137468; BH-adjusted *p* = 0.1925). We did not observe a statistically significant difference in the percentage of this cell subpopulation in the PB of patients and the control group (*p* = 0.14231; BH-adjusted *p* = 0.1992; 95% CI [−14.00, 18.19]).

Moreover, we found a significantly higher percentage of DNAM-1^+^CD56^+^CD16^+^CD3^−^ cells in the PF than in the PB of OC patients (median 70.34% vs. 58.29%; *p* = 0.033945; BH-adjusted *p* = 0.0679; 95% CI [−9.41, 27.94]) and higher than in the TT (*p* = 0.483840; BH-adjusted *p* = 0.553; median PF vs. TT totals 70.34% vs. 54.62%, 95% CI [−29.62, 29.03]) and the PB of healthy donors (median 70.34% vs. 54.99%; 95% CI [−29.08, 7.82]); however, the differences were not statistically significant (*p* = 0.08081; BH-adjusted *p* = 0.9428) (Figure 3D). We did not observe a statistically significant difference in the percentage of this cell subpopulation in the PB between patients and the control group (*p* and BH-adjusted *p* = 0.906376; 95% CI [−19.72, 25.80]).

### 3.3. Concentration of Soluble Forms of PD-1, TIGIT, and DNAM-1 Receptors in Ovarian Cancer Environments

**Figure 4:**
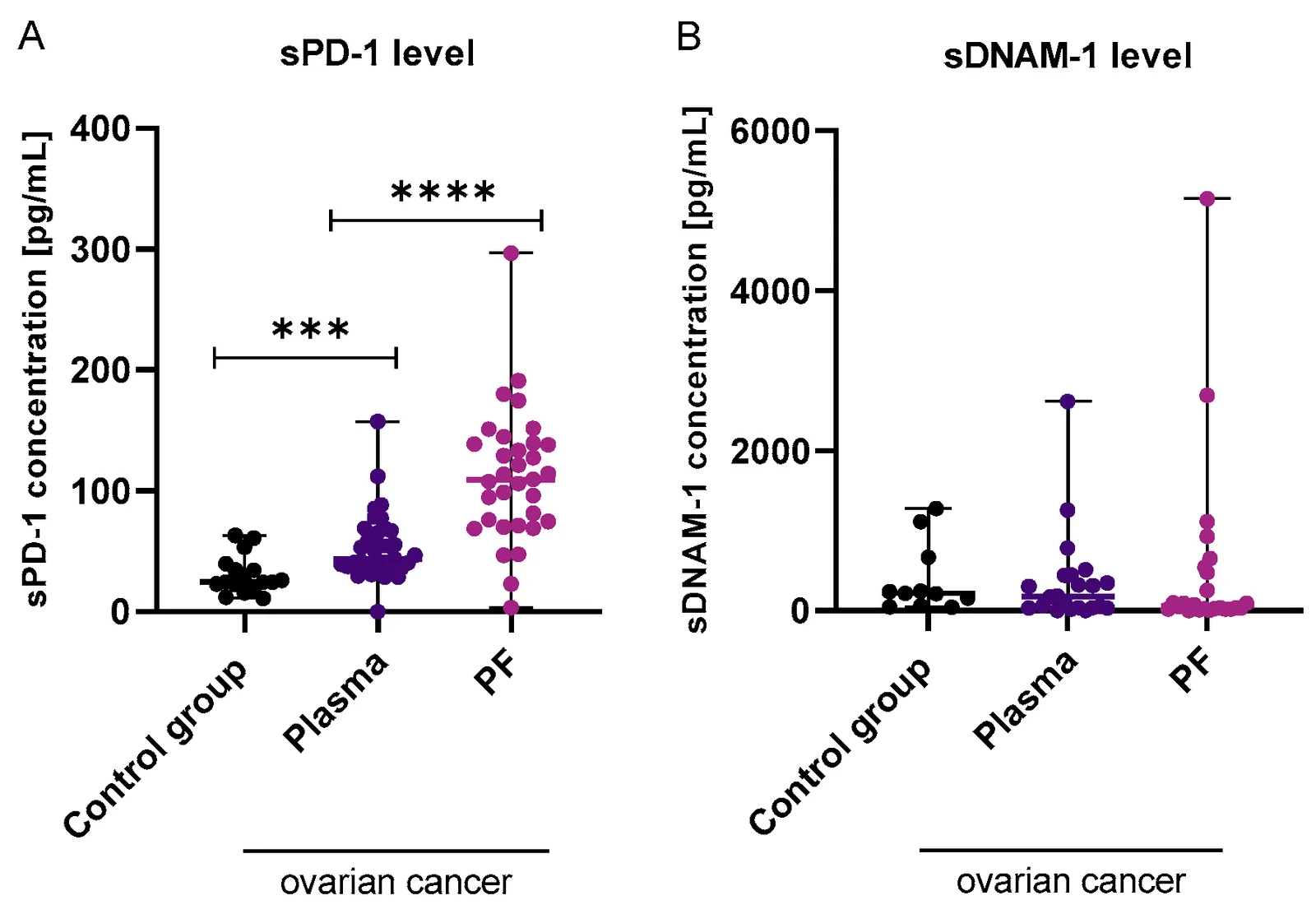
Concentrations of soluble form of programmed death receptor 1 (sPD-1) (**A**) and soluble form of DNAX accessory molecule-1 (sDNAM-1) (**B**) in the plasma and peritoneal fluid (PF) of ovarian cancer (OC) patients compared to the plasma of healthy blood donors. Paired plasma and peritoneal fluid samples were compared using the Wilcoxon signed-rank test, while independent groups were compared using the Mann–Whitney U test. Median values with the following marks indicate statistically significant differences: *** *p* < 0.001, **** *p* < 0.0001.

Our analysis revealed differences in the concentrations of sPD-1 and sTIGIT in the plasma and PF of OC patients and in the plasma of healthy donors. We observed the highest level of sPD-1 in PF, which was significantly higher than that in the plasma of OC patients (*p* = 0.000001; BH-adjusted *p* = 0.000008; median 109.54 pg/mL vs. 43.80 pg/mL; 95% CI [37.44, 76.96]). We also detected a significantly higher level of sPD-1 in the plasma of OC patients than in that of the control group (*p* = 0.00022; BH-adjusted *p* = 0.00088; median 43.80 pg/mL vs. 24.97 pg/mL; 95% CI [−14.21, 20.88]) (Figure 4A).

The results revealed the highest concentration of sDNAM-1 in the plasma of healthy blood donors, which was higher than that in the plasma (*p* = 0.522217; BH-adjusted *p* = 0.5968; median 215.65 pg/mL vs. 177.74 pg/mL; 95% CI [−29.45, −10.57]) and PF (*p* = 0.13628; BH-adjusted *p* = 0.218048; median 215.65 pg/mL vs. 61.29 pg/mL; 95% CI [−34.20, 21.22]) of OC patients. Nevertheless, we did not observe statistically significant differences (Figure 4B). These findings should be interpreted in the context of the demographic differences between the patient and healthy control groups described above.

Notably, the differences in the number of patients included in the sTIGIT concentration analysis resulted from the difficulty of meeting the predefined measurement range, as the values were highly dispersed. Some samples were excluded because their sTIGIT levels in plasma or PF were too low to be detected within the assay range, whereas others exceeded the upper limit of quantification.

Moreover, we detected the highest concentration of sTIGIT in the plasma of the OC, which was significantly higher than that in the plasma of the control group (*p* = 0.01546; BH-adjusted *p* = 0.04123; median 4.58 pg/mL vs. 1.44 pg/mL; 95% CI [−10.99, −0.58]) and higher than that in the PF (median 4.58 pg/mL vs. 1.56 pg/mL). Notably, the number of samples included in the sTIGIT concentration analysis differed between groups because some measurements fell outside the validated assay range. Samples with sTIGIT concentrations below the lower limit of detection (LLOD) could not be reliably quantified and were therefore excluded from the analysis, while samples exceeding the upper limit of detection (ULOD) were similarly excluded. Given the variability in sTIGIT concentrations and the presence of measurements outside the validated assay range, the presented findings should be interpreted as exploratory. The percentages of samples below the LLOD and ULOD in each study group are presented in Table 2.

### 3.4. Percentages of NK Cells with PD-1, TIGIT or DNAM-1 Expression in Patients with Different Clinical Manifestations of OC

We subsequently evaluated the clinical relevance of NK cells with PD-1, TIGIT, or DNAM-1 expression in OC patients. The percentage of these cell subpopulations was correlated with particular OC clinicopathological features—i.e., FIGO stage (I–II vs. III–IV), grade (II vs. III), and Kurman and Shih tumor type (type I vs. type II)—in comparison to their distribution in the PB of healthy donors. These findings should be interpreted in the context of the demographic differences between the patient and healthy control groups described above.

**Figure 5:**
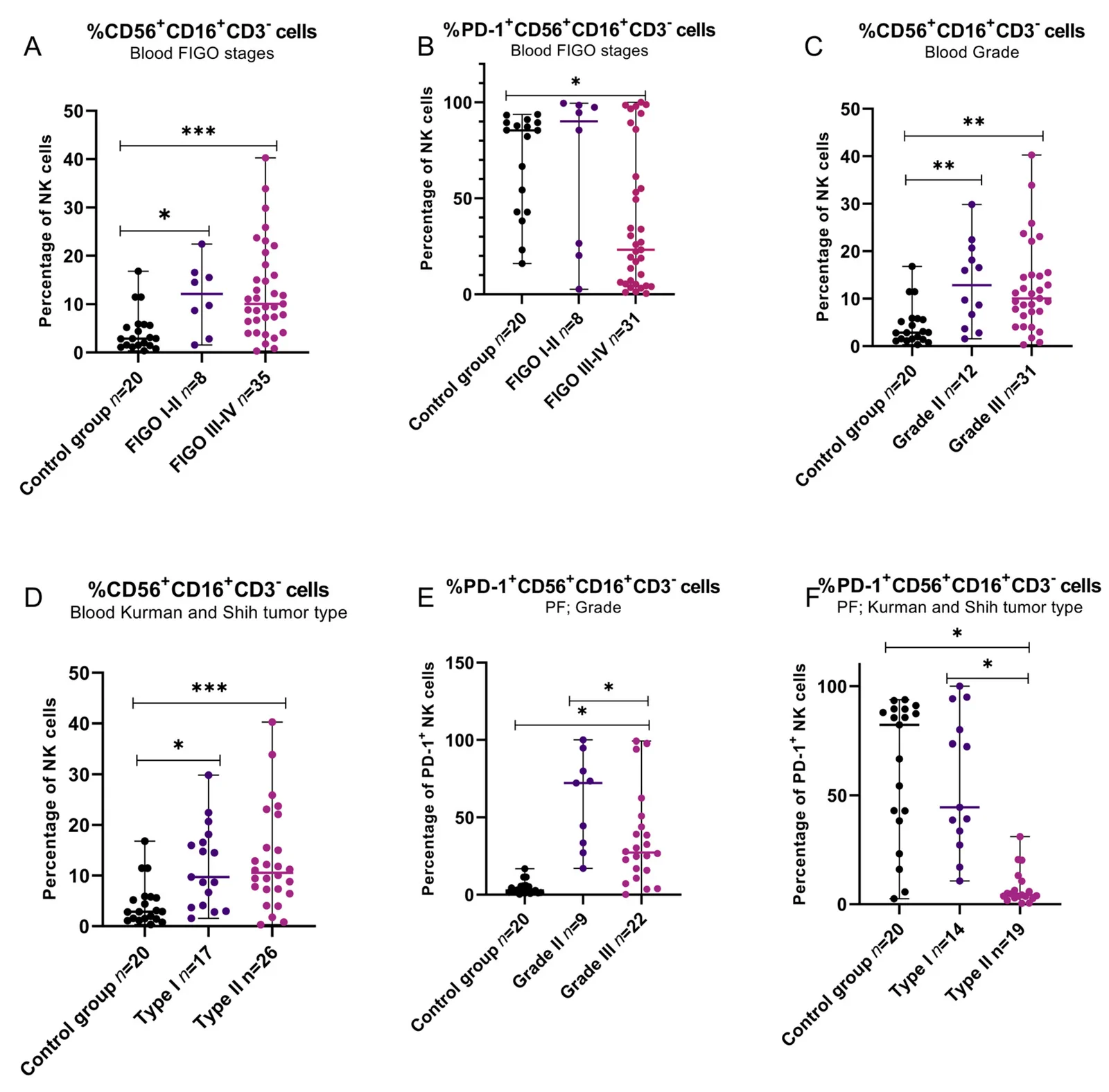
Distribution of NK (CD56^+^CD16^+^CD3^−^) cells across different FIGO stages (**A**,**B**), grading (**C**), and ovarian cancer (OC) types according to Kurman and Shih (**D**) in the peripheral blood (PB) of OC patients. NK (CD56^+^CD16^+^CD3^−^) cells with different PD-1 expression in different grades (**E**) and OC types according to Kurman and Shih (**F**) in the peritoneal fluid (PF) of OC patients in comparison with the PB of healthy donors. Independent groups were compared using the Mann–Whitney U test. Median values with the following marks indicate statistically significant differences: * *p* < 0.05, ** *p* < 0.01, *** *p* < 0.001.

We found that the percentage of CD56^+^CD16^+^CD3^−^ cells in the PB of OC patients was significantly higher in advanced FIGO stages (III–IV) than in the PB of the control group (*p* = 0.00016; BH-adjusted *p* = 0.00367; 95% CI [−10.24, −3.33]) (Figure 5A), and it was lower in advanced FIGO stages (III/IV) than in early FIGO stages (I/II). Nevertheless, the difference was not statistically significant (*p* and BH-adjusted p totaling 0.9627; 95% CI [−6.50, 7.25]) (Figure 5A). We observed the highest percentage of this cell subpopulation in PB of patients with early FIGO stages (I/II), and it was significantly higher than in the control group (*p* = 0.00013; BH-adjusted *p* = 0.0051; 95% CI [−13.08, −1.23]).

Moreover, we showed the lowest percentage of PD-1^+^CD56^+^CD16^+^CD3^−^ cells in the PB of OC patients with advanced FIGO stages (III–IV), and it was significantly lower than the control group (*p* = 0.04249; BH-adjusted *p* = 0.2732; 95% CI [0.89, 55.54]) (Figure 5B). There was a significantly lower percentage of CD56^+^CD16^+^CD3^−^ cells in the PB of the control group than in group of patients with grade II disease (*p* = 0.003292; BH-adjusted *p* = 0.07431; 95% CI [−15.03, −2.16]) (Figure 5C) and those with grade III disease (*p* = 0.00345; BH-adjusted *p* = 0.01554; 95% CI [−9.70, −3.27]) (Figure 5C). We detected the lowest percentage of CD56^+^CD16^+^CD3^−^ cells in the PB of control group in comparison to patients with type II tumors according to Kurman and Shih (*p* = 0.0016.09; BH-adjusted *p* = 0.03621; 95% CI [−10.07, −3.62]) (Figure 5D), and patients with type I tumors (*p* = 0.000483; BH-adjusted *p* = 0.21742; 95% CI [−12.87, −2.16]) (Figure 5D).

We also showed that the percentage of PD-1^+^CD56^+^CD16^+^CD3^−^ cells was significantly higher in the PF of patients with grade II OC than in those with grade III (*p* = 0.0348; BH-adjusted *p* = 0.2237; 95% CI [−55.69, −0.64]) (Figure 5E). Moreover, we found that the percentages of this cell subpopulation in PF were significantly higher in OC patients with grade III than in healthy blood donors (*p* = 0.03311; BH-adjusted *p* = 0.2483; 95% CI [3.67, 56.94]) (Figure 5E). We observed that the percentage of PD-1^+^CD56^+^CD16^+^CD3^−^ cells was lower in PF of patients with type II tumors in comparison to control group (*p* = 0.023586; BH-adjusted *p* = 0.2123; 95% CI [−4.69, 49.12]) (Figure 5F) and lower than in the group of patients with type I tumors (*p* = 0.032217; BH-adjusted *p* = 0.2071; 95% CI [−19.41, 17.49]) (Figure 5F). There was no other significant disparity in the percentages of NK cells and PD-1-, TIGIT-, or DNAM-1-positive NK cells in PB, PF, or TT in the different clinicopathological features of patients with OC (*p* > 0.05).

### 3.5. Relationships Between the Clinicopathological Characteristics of OC Patients and the Percentages of NK Cells with PD-1, TIGIT, or DNAM-1 Expression in OC Environments

**Figure 6:**
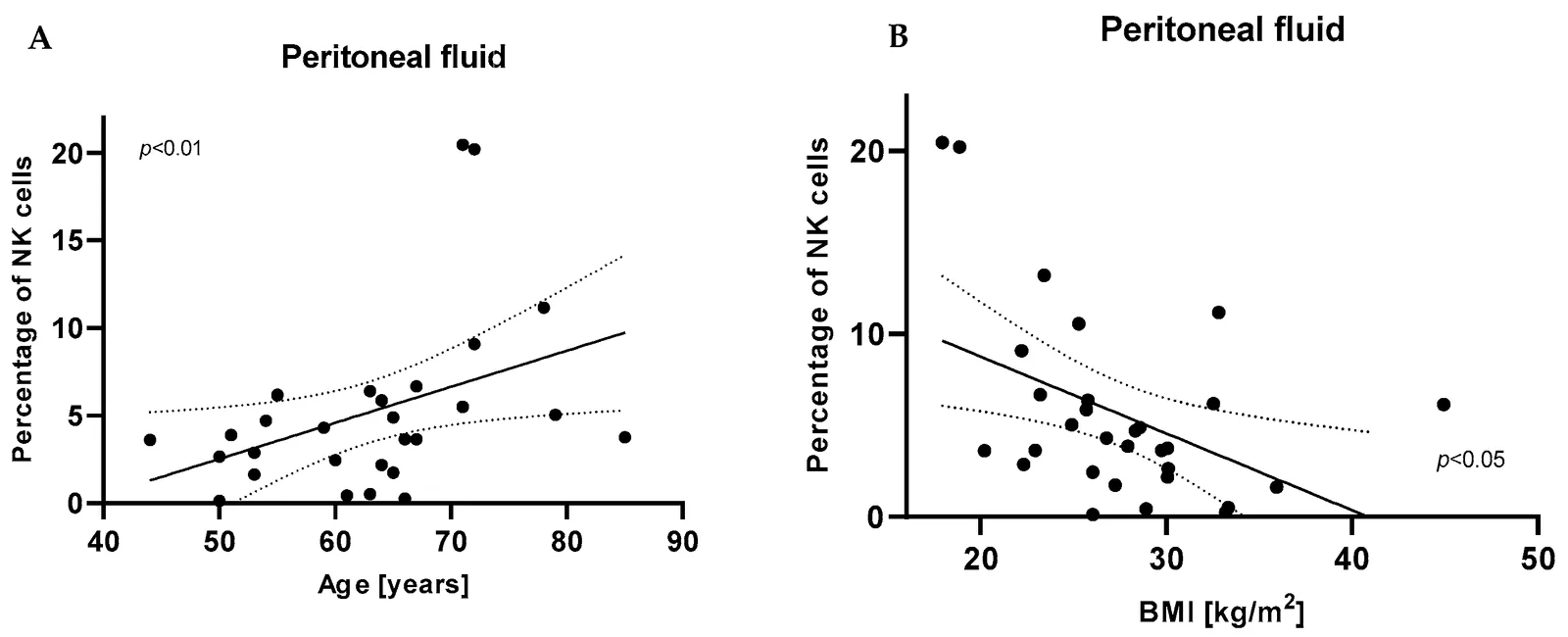
Positive correlation between the percentage of NK (CD56^+^CD16^+^CD3^−^) cells in peritoneal fluid (PF) and age (*p* < 0.01; Spearman’s R = 0.471; t(N − 2) = 2.777) in ovarian cancer (OC) patients (**A**). Negative correlation between the percentage of NK (CD56^+^CD16^+^CD3^−^) cells and body mass index (BMI) (*p* < 0.05; Spearman’s R = −0.437; t(N − 2) = −2.569) in ovarian cancer (OC) patients (**B**). The correlations were evaluated using Spearman’s rank correlation analysis.

In the next step, we assessed the relationships between the clinicopathological characteristics of patients with OC and the percentages of CD56^+^CD16^+^CD3^−^ cells and CD56^+^CD16^+^CD3^−^ cells with PD-1, TIGIT, or DNAM-1 expression in three different OC environments. We detected a positive relationship between the percentage of CD56^+^CD16^+^CD3^−^ cells in the PF and the age of OC patients (Spearman’s R = 0.471; t(N − 2) = 2.777, *p* = 0.009853; 95% CI [−0.149, 0.487]) (Figure 6A). Conversely, there was a negative association between the percentage of CD56^+^CD16^+^CD3^−^ cells in the PF and BMI in OC patients (Spearman’s R = −0.437; t(N − 2) = −2.569, *p* = 0.015802; 95% CI [−0.629, 0.03]) (Figure 6B).

There were no other significant relationships between the percentages of NK cells and NK cells with PD-1, TIGIT, or DNAM-1 expression in PB, PF, and among tumor-infiltrating cells and the clinicopathological characteristics of OC patients—i.e., FIGO stage, grade, age, type of tumor according to Kurman and Shih, menopausal status, and HE4 and CA125 concentrations (*p* > 0.05).

## 4. Discussion

Despite advancements in ovarian cancer treatment, the five-year survival of OC patients is still poor. Recently published studies, including both fundamental studies and clinical trials, have focused on the application of ICIs, mainly anti-PD-1/PD-L1, in OC treatment [19,38,39]. Notably, the role and clinical implications of coexpressed ICPs, both coinhibitory (TIGIT) and costimulatory (DNAM-1), on effector immune cells in OC are still unexplored. Thus, investigating the role of not only the PD-1/PD-L1/PD-L2 axis but also other coexpressed ICPs is necessary to develop effective tools to treat OC patients.

NK cells play a predominant role in immune surveillance. Their activity is controlled by a balance between activating and inhibitory receptors, which allows them to selectively eliminate tumor cells that display activating ligands and evade those with strong inhibitory signals. The effectiveness of NK cells in OC patients is inhibited by immunosuppressive factors within the TME [40,41]. We aimed to characterize the NK cell immunophenotype across different OC environments, specifically the balance between inhibitory (PD-1, TIGIT) and activating (DNAM-1) receptors. Thus, we evaluated the expression of PD-1, TIGIT, and DNAM-1 on NK cells in three different environments (PB, PF, TT) of OC patients compared with healthy blood donors. Our study demonstrated alterations in the TIGIT/CD155/DNAM-1 and PD-1/PD-L1/PD-L2 axes and changes in NK cell immunophenotype both systemically and within the ovarian TME.

Our findings revealed altered percentages of NK cells with PD-1, TIGIT, and DNAM-1 expression across different environments in OC patients. Similarly to Maas et al., we observed an immunophenotype shift within the NK cells in favor of an inhibitory shift in local environments in comparison to PB [40]. The increased expression of inhibitory receptors—i.e., PD-1 among tumor-infiltrating cells and TIGIT in PF—may indicate the presence of local immunosuppression that could contribute to reduced NK cell activity in OC. These results align with previous reports indicating that alterations in the TME are potentially associated with changes in the balance of activating and inhibitory receptors on NK cells, which may be relevant for future immunotherapy research [25,40,42]. Moreover, the upregulation of TIGIT and DNAM-1 expression on NK cells in PF may reflect altered regulation of the TIGIT/CD155/DNAM-1 axis within the local environment. Given that TIGIT and DNAM-1 share common ligands (i.e., CD155), these findings warrant further investigation into the potential functional consequences of their coexpression in OC. Notably, according to Pounds et al., there is a relationship between the increased percentage of NK cells with DNAM-1 expression and poor survival of OC patients [43].

The present study revealed altered concentrations of soluble ICPs in OC patients. Specifically, the sPD-1 concentration was the highest in PF, suggesting its local accumulation in the TME. Moreover, significantly higher concentrations of sPD-1 and sTIGIT were detected in the plasma of OC patients than in that of healthy controls, suggesting alterations in systemic immune status. Although the differences in the concentrations of sDNAM-1 in different OC environments and the control group did not reach statistical significance, we observed a consistent trend toward its highest concentration in the plasma of healthy donors, which may reflect local immunosuppression. Previous reports on the immunosuppressive role of soluble ICPs in the TME have shown that OC may promote immune evasion through increased concentrations of inhibitory signals (sPD-1, sTIGIT) and decreased activating signals (sDNAM-1) [42,44,45]. However, further mechanistic studies are required to confirm the functional relevance of these alterations.

Changes in NK cell immunophenotypes can also be associated with the clinical features of OC. Thus, we assessed the clinical relevance of NK cells expressing PD-1, TIGIT, and DNAM-1 in OC patients. Compared to the control group, a higher percentage of NK cells was observed in the PB of OC patients with advanced stages of disease (FIGO stage III/IV), both grades II and III, and tumor types I and II according to Kurman and Shih. These findings indicate an association between the percentage of circulating NK cells and clinical indicators of disease progression and tumor aggressiveness. The increased percentage of PD-1^+^ NK cells in PF in grade II of OC compared with that in the control group and their higher percentage in type I compared with type II of OC may reflect differences in NK cell distribution and immunophenotypic composition across disease stages and OC subtypes. Similar to our findings, Greppi et al. reported an increased percentage of PD-1^+^ NK cells in the peritoneal fluid of high-grade serous ovarian cancer patients, while this cell subpopulation is rarely detected in healthy blood donors. Moreover, the increased percentage of these cells was related to poor overall survival, which highlights the clinical and prognostic significance of PD-1^+^ NK cells [46].

We further investigated the relationships between the clinicopathological features of OC patients and the percentages of NK cells or NK cells with PD-1, TIGIT, or DNAM-1 expression in different OC environments. Similar to other authors [47,48,49,50], we reported a positive correlation between the percentage of CD56^+^CD16^+^CD3^−^ cells in ascites and the age of OC patients. Reports in the literature indicate that aging can be associated with increased NK cell percentage but reduced cytotoxic function [48,50]. Moreover, Fraser et al. reported that the antitumor activity of NK cells in peritoneal fluid is inhibited via CA125 [51]. Additionally, the percentage of CD56^+^CD16^+^CD3^−^ cells in the PF negatively correlated with the BMI of OC patients. Many studies [52,53,54,55] have shown that obesity is related to altered NK cell numbers and their impaired cytotoxic functions, including cytokine expression, which leads to weakened immunosurveillance.

Notably, not all OC patients present with ascites or PF, which may limit the generalizability of PF-based findings to the entire OC population. However, PF represents a clinically relevant component of the TME in a substantial proportion of patients with advanced disease and provides important insight into local immune regulation in OC. The relatively limited sample size represents a key limitation of this study. Due to the limited availability of biological material, particularly for analyses of specific compartments and cellular populations, some comparisons were based on relatively small subgroup sizes. This was particularly relevant for analyses of tumor-infiltrating NK cell populations. Therefore, these findings should be considered preliminary and interpreted primarily as descriptive or exploratory observations rather than definitive inferential evidence. Similarly, the differences in the number of patients included in the sTIGIT concentration analysis resulted from the difficulty of meeting the predefined measurement range, as the values were highly dispersed. Some samples were excluded because their sTIGIT levels in plasma or PF were too low to be detected within the assay range, whereas others exceeded the upper limit of quantification. Further studies involving larger cohorts and greater availability of biological material are warranted to validate these observations. Another limitation of this study is the absence of disease control groups, such as patients with benign inflammatory gynecological conditions (e.g., endometriosis or pelvic inflammatory disease), which would allow better assessment of the specificity of NK cell ICP alterations for OC. The healthy control group consisted exclusively of premenopausal women, whereas the patient cohort included both premenopausal and postmenopausal women. Differences in age, menopausal status, and BMI may therefore represent potential confounding factors in comparisons between groups. Moreover, peripheral blood samples from healthy donors served as a baseline comparator for circulating immune cells only and did not provide a direct healthy reference for the PF or TT compartments.

The intricate interplay between the TIGIT/CD155/DNAM-1 axis and the PD-1/PD-L1/PD-L2 pathway underscores the complexity of restoring NK cells’ effector functions.

## 5. Conclusions

Ovarian cancer is associated with alterations in NK cell distribution and the expression of inhibitory and activating receptors across tumor and peripheral compartments, reflecting changes in the immune landscape of the TME and systemic circulation. In our study, we observed an increased frequency of PD-1^+^ NK cells among tumor-infiltrating immune cells and an enrichment of TIGIT^+^ NK cells in PF, indicating differential expression patterns of inhibitory receptors across these compartments. In parallel, we detected an increased percentage of DNAM-1^+^ NK cells in peritoneal fluid.

In addition, concentrations of sTIGIT in plasma and sPD-1 in both plasma and peritoneal fluid were higher in patients with OC than in healthy controls. Finally, NK cell immunophenotypes were associated with selected clinicopathological parameters of OC. The frequency of NK cells and PD-1^+^ NK cells showed associations with disease-related features, whereas NK cell percentages in peritoneal fluid were associated with patient age and BMI, indicating that these clinical variables may be relevant to NK cell distribution in the studied cohort.

## Figures and Tables

**Table 1:** Clinicopathological characteristics of OC patients.

The Clinical Features	OC Patients (*n* = 56)
FIGO stage, *n* (%)	
Early (FIGO stages I–II)	11 (19.64%)
Advanced (FIGO stages III–IV)	45 (80.36%)
Grading (histological differentiation), *n* (%)	
Intermediate grade (G2)	17 (30.36%)
Low grade (G3)	39 (69.64%)
The OC classification according to Kurman and Shih, *n* (%)	
Type I	21 (37.5%)
Type II	35 (62.5%)
Menopausal Status	
Before menopause	10 (17.86%)
After menopause	46 (82.14%)
CA125 median, range (U/mL)	491.00 (14.96–8766)
HE4 median, range (pmol/L)	583 (45.3–12,114)
Age (median), years (range)	61 (29–85)
Body mass index (BMI) range, median (kg/m^2^)	27.73 (17.94–44.92)

**Table 2:** sTIGIT measurements below the lower limit of detection and above the upper limit of detection across study groups.

	Plasma of Control Group (%)	Plasma of Ovarian Cancer Patients (%)	Peritoneal Fluid of Ovarian Cancer Patients (%)
sTIGIT lower limit of detection (LLOD)	2/16 (12.5%)	3/31 (9.68%)	27/31 (87.1%)
sTIGIT upper limit of detection (ULOD)	4/16 (25%)	10/31 (32.26%)	0/31 (0%)
Total	6/16 (37.5%)	13/31 (41.94%)	27/31 (87.1%)

## Data Availability

The datasets generated and/or analysed during the current study are available in the RepOD (Repository for Open Data) (https://repod.icm.edu.pl/dataset.xhtml?token=7bf6968a-bd64-4f72-8676-5e90b8a5be95, accessed on 13 October 2026).

## Abbreviations

The following abbreviations are used in this manuscript:

BH	Benjamini–Hochberg correction
BMI	Body mass index
CD	Cluster of differentiation
DNAM-1	DNAX accessory molecule-1
FDA	Food and Drug Administration
FDR	False discovery rate
FIGO	International Federation of Gynecology and Obstetrics
HGSOC	High-grade serous ovarian cancer
ICI	Immune checkpoint inhibitor
ICP	Immune checkpoint protein
LLOD	Lower limit of detection
MNC	Mononuclear cell
NK cells	Natural killer cells
OC	Ovarian cancer
PARP	Poly(ADP-ribose) polymerase
PARPi	Poly(ADP-ribose) polymerase inhibitor
PB	Peripheral blood
PD-1	Programmed cell death protein 1
PD-L1	Programmed death ligand 1
PD-L2	Programmed death ligand 2
PF	Peritoneal fluid
sDNAM-1	Soluble DNAX accessory molecule-1
sPD-1	Soluble programmed cell death protein 1
sTIGIT	Soluble T cell immunoreceptor with immunoglobulin and ITIM domains
TIGIT	T cell immunoreceptor with immunoglobulin and ITIM domains
TME	Tumor microenvironment
TT	Tumor tissue
ULOD	Upper limit of detection
VEGFi	Vascular endothelial growth factor inhibitor
